# The regulation and mechanism of the cAMP-PKA pathway on PTSD-like behaviors exacerbated by alcohol exposure

**DOI:** 10.3389/fphar.2025.1592187

**Published:** 2025-05-16

**Authors:** Shuang Zhao, Wei Zhao, Ziqi Wang, Xiaofei Chen, Fangjiao Zong, Hanting Zhang

**Affiliations:** Department of Pharmacology, Qingdao University School of Pharmacy, Qingdao, China

**Keywords:** cAMP-PKA signaling, alcohol exposure, PTSD, fear memory extinction, synaptic plasticity

## Abstract

**Background:**

Alcohol use disorder (AUD) and post-traumatic stress disorder (PTSD) exhibit a significant degree of comorbidity. Nevertheless, the specific effects and underlying mechanisms by which alcohol, as a risk factor, contributes to the development of PTSD-ike phenotypes remain poorly understood. Both chronic alcohol consumption and exposure to traumatic stress can lead to synaptic damage in the hippocampus, potentially serving as a neurobiological basis for the exacerbation of PTSD induced by alcohol.

**Methods:**

In this study, an animal model was established by allowing mice to voluntarily consume alcohol for 2 weeks, followed by exposure to a single prolonged stress combined with foot shock (SPS&FS). Subsequently, the mice received an intraperitoneal injection of rolipram (1 mg/kg), and behavioral, biochemical, and morphological analyses were performed.

**Results:**

The findings revealed that individuals with early alcohol exposure exhibited more pronounced deficits in fear extinction during the fear extinction task (FET) and displayed higher levels of anxiety-like behavior in both the open field test (OFT) and the elevated plus maze test (EPM). Activation of cAMP-PKA signaling enhanced the downregulation of brain-derived neurotrophic factor (BDNF) and tyrosine kinase receptor B (TrkB), upregulated the expression of PSD95, synaptophysin, AMPA, and NMDA receptor subtypes, and reversed the impairment of CA1 synaptic function and dendritic structure in the hippocampus.

**Conclusion:**

Activation of the cAMP-PKA pathway facilitated fear extinction in PTSD mice with early alcohol exposure, alleviated anxiety-like behavior, attenuated symptoms of AUD following ethanol relapses. These findings suggest that modulating hippocampal synaptic plasticity by activating the cAMP-PKA pathway may represent a promising therapeutic approach for attenuating alcohol-exacerbated PTSD-like behaviors.

## 1 Introduction

Post-traumatic stress disorder (PTSD) is a complex psychiatric condition triggered by traumatic events, characterized primarily by persistent intrusive fear memories that are difficult to extinguish (Maercker et al., 2022; Bryant et al., 2023; Yanpallewar et al., 2022; Messent, 2013). This leads to the long-term maintenance of anxiety-related symptoms in PTSD patients (Wen et al., 2022). Currently, enhancing fear extinction is a pivotal focus in PTSD treatment research; however, the precise neurobiological mechanisms underlying this process have yet to be fully elucidated. Long-term alcohol exposure has been identified as a significant risk factor for the development of PTSD (Logrip et al., 2012). Clinical studies conducted on military personnel revealed that the prevalence of PTSD was 9.8%, whereas the prevalence of PTSD among individuals with a history of alcohol consumption was significantly higher, reaching 27.5% (Stein et al., 2017). Clinical evidence demonstrates that alcohol exposure impairs the extinction of fear memories, thereby exacerbating negative emotional states such as anxiety-like behaviors and promoting the development of PTSD (McDevitt-Murphy et al., 2017; Wen et al., 2022). This impairment in emotional regulation further reinforces an individual’s psychological dependence on alcohol, forming a “negative reinforcement” reward mechanism that ultimately leads to excessive drinking behavior and contributes to the development of AUD (Weiss et al., 2021). This creates a vicious cycle between PTSD and alcohol use disorder (AUD), severely compromising patients’ mental health and social functioning (Leeies et al., 2010).

Prolonged exposure (PE) therapy has been developed to mitigate trauma-related fear and anxiety in patients with PTSD through the process of fear extinction (Back et al., 2019; Hunt et al., 2023). The amygdala, hippocampus, and prefrontal cortex are all involved in the regulation of fear memories (Izquierdo et al., 2016). Among these regions, the hippocampus is considered particularly critical for fear memory extinction (Lacagnina et al., 2019). Neuroimaging studies consistently demonstrate significantly reduced hippocampal activation patterns in individuals with severe AUD and those diagnosed with PTSD (Wang Z. et al., 2023). Additionally, a notable reduction in hippocampal volume has been observed, which may be associated with impaired synaptic plasticity in this region (Harnett et al., 2020; Garcia, 2002). Consequently, enhancing synaptic function in the hippocampus may improve the efficacy of extinction learning, which is crucial for alleviating the symptoms of PTSD.

Brain-derived neurotrophic factor (BDNF) is a crucial regulator of synaptic plasticity in the brain (Peters et al., 2010). Activation of BDNF and its receptor tyrosine kinase receptor B (TrkB) can mitigate stress-induced impairments in fear memory extinction, thereby ameliorating anxiety-like behaviors associated with PTSD (Shimoda et al., 2024; Sur and Lee, 2022). The BDNF-mediated effect is closely linked to glutamatergic neurotransmission, particularly through the modulation of AMPA and NMDA receptors, which play essential roles in enhancing synaptic plasticity and regulating learning and memory processes, including hippocampal-dependent fear memory extinction (Park et al., 2021; Radulovic et al., 2019). Furthermore, the levels of PSD95 are strongly correlated with long-term potentiation (LTP). Its upregulation may facilitate synaptic remodeling, thereby potentially promoting fear memory extinction (Suzuki et al., 2018; Li et al., 2018; Ziółkowska et al., 2023). Meanwhile, synaptophysin, a presynaptic vesicular membrane protein, exhibits dynamic expression patterns that are likely to reflect alterations in synaptic transmission efficacy. These changes may contribute to either the suppression or extinguishing of fear memories (Cousin, 2021; Popova et al., 2014).

Cyclic adenosine monophosphate (cAMP), a pivotal second messenger, facilitates LTP by activating protein kinase A (PKA) and is essential for synaptic transmission, neuronal excitability, neuroplasticity, and neuroprotection (Shahoha et al., 2022; Heckman et al., 2018). These findings suggest that pharmacological modulation of hippocampal synaptic function via PKA signaling pathway activation may represent a promising therapeutic strategy for alcohol-induced PTSD-like behaviors.

The present study aims to investigate the neural mechanisms by which alcohol exposure exacerbates PTSD-like behaviors and to further validate whether activating the cAMP-PKA signaling pathway can restore synaptic plasticity impairment, thereby ameliorating PTSD and AUD-associated behaviors. By elucidating this mechanism, this study will provide a theoretical foundation for developing novel intervention strategies for comorbid PTSD and AUD.

## 2 Materials and methods

### 2.1 Animals

The experimental protocol employed adult male C57BL/6J mice (purchased from Beijing Weitong Lihua Animal Laboratory Co., Ltd.) aged between 6 and 8 weeks, with body weights ranging from 20 to 25 g. Environmental parameters in the animal facility were strictly controlled, with ambient temperature maintained at 24°C ± 2°C and relative humidity 40%–50%. A standardized 12 h light/dark cycle was implemented. Individual housing was provided for each mouse, ensuring *ad libitum* access to food and water supply. Animal procedures were approved by the Committee of Animal Experimental Ethics of Qingdao University, China (approval number: QDU-AEC-2025099).

### 2.2 SPS&FS procedure

The experimental protocol for inducing PTSD-like behavior in murine models was conducted using a standardized combination of prolonged stress and plantar electrical stimulation, as previously described (Xi et al., 2023; Stein et al., 2017; Xi et al., 2021; 2023; Lou et al., 2022; Wang et al., 2022). The procedure was executed in the following sequence: Initially, mice were subjected to confinement in a restraint apparatus for 2 h, after which they were immediately transferred to an acrylic chamber (50 cm × 30 cm) containing water maintained at a temperature of 22°C–24°C for a 20 min forced swim session. Subsequently, the mice were exposed to ether vapor until the onset of unconsciousness (duration <1 min), and this procedure was repeated twice consecutively. After an additional 15 min rest interval, the subjects were administered a single electric shock (2 mA, 2 s) within a designated apparatus and returned to their respective home cages.

### 2.3 Two-bottle choice test (2-BC)

A continuous two-bottle choice paradigm was employed to evaluate murine ethanol consumption patterns over 24 h (Wei et al., 2024). The mice were individually housed in cages equipped with two plastic bottles, offering a choice between an ethanol solution and water. The rodents were granted unrestricted access to both beverages. The alcohol concentration systematically increased from 10% to 15% and subsequently stabilized at 20%. To mitigate potential location-based preferences, the water bottle positions were alternated every 24 h. Both ethanol and water were replenished daily at fixed times to ensure consistency in fluid availability and concentration.

### 2.4 Conditioned place preference (CPP)

The ethanol-induced conditioned place preference (CPP) experiment was conducted using a three-compartment environmental conditioning apparatus, which primarily comprised two distinct chambers (Lei et al., 2024; Lu et al., 2017; Pati et al., 2019; Sharifi et al., 2022; Potula et al., 2023). One of these is black with a relatively smooth floor and is designated as the unconditioned side. The opposing chamber featured a black-and-white striped pattern with a circular texture on the floor and was designated as the conditioned side. These two compartments were delineated using a smaller intermediary box. Throughout the training phase, an isolation door was inserted into the middle compartment to partition the two chambers, thereby creating an entirely separate environment. The experimental protocol for the CPP was segmented into three stages: baseline preference assessment (day 1), conditioning phase (days 2–9), and post-conditioning test (day 10).

During the pre-conditioning phase (day 1), the divider was removed to permit unrestricted access to both chambers, allowing mice to freely explore the apparatus for 20 min. Behavioral tracking was performed using a camera recording system to quantify the time spent in each chamber, enabling the calculation of baseline preference scores for both conditioned and unconditioned sides. Animals exhibiting a preference of >800 s for either compartment were excluded from subsequent statistical analysis, to ensure balanced baseline preferences (Wang J. et al., 2023).

The conditioning phase (days 2–9) consisted of eight consecutive days of training. On alternate days (days 2, 4, 6, and 8), the mice received intraperitoneal injections of saline and were confined to the unconditioned chamber for 30 min. Conversely, on the intervening days (days 3, 5, 7, and 9), the mice were administered ethanol (2 g/kg, i.p.) and restricted to the conditioned chamber for an equivalent duration. This alternating schedule resulted in four pairings for each treatment condition. The control animals received saline injections during all the conditioning sessions.

On the post-conditioning test day (day 10), the isolation doors on both sides were removed, and the mice were allowed unrestricted access to both chambers for 20 min. The time spent in each chamber was recorded to assess the place’s preference.

To prevent interference from olfactory cues, the apparatus was thoroughly cleaned with a 75% ethanol solution between trials, a blower device was used to accelerate the dissipation of alcohol odor. Behavioral testing began after 30 min to ensure alcohol odor dissipation and prevent experimental interference.

$$\text{Preference score}=\left(\text{Conditional time}-\text{unconditional time}/\text{Conditional time}+\text{unconditional time}\right)$$

### 2.5 Experimental procedures and drug administration

Following the procurement of experimental animals, all mice underwent a 7-day acclimatization period in the housing facility under standard conditions. The mice were equally divided into five groups (n = 9 per group): Control, Control + EtOH, SPS & FS, SPS & FS + EtOH, and SPS & FS + EtOH + ROL. Control, SPS & FS, SPS & FS + EtOH, and SPS & FS + EtOH + ROL for the assessment of behavioral, Western blot, and Golgi staining; Specifically, six mice were randomly selected from each group for Western blot analysis. The remaining three mice in each group were preserved with intact whole brains for Golgi staining. To ensure sufficient sample size and reliable Golgi staining results, an additional two mice per group were included under identical experimental conditions specifically dedicated to Golgi staining analysis (n = 5 per group). Control, Control + EtOH, SPS & FS + EtOH, SPS & FS + EtOH + ROL for the detection of 2-BC and CPP.

For pharmacological intervention, mice in the treatment group received daily intraperitoneal injections of rolipram (1 mg/kg; Cat: HY-16900; MCE), whereas control animals were administered an equivalent volume of vehicle solution following the same injection protocol.

### 2.6 Behavioral tests

In our animal behavioral experiments, we strictly adhered to a double-blind protocol to minimize potential experimenter bias and ensure objective data collection.

#### 2.6.1 Open field test (OFT)

The OFT was conducted following established protocols (Zhao et al., 2024). The open field test was conducted using a white cube-shaped apparatus (50 cm × 50 cm × 50 cm). Each mouse was placed in a corner at the start of the test and recorded for 10 min using a camera located above the box. To eliminate potential olfactory cues between trials, the apparatus was thoroughly cleaned with a 75% ethanol solution. Behavioral data were analyzed using the VisuTrack automated tracking system (Xin Ruan Information Technology Co., Ltd., Shanghai, China).

#### 2.6.2 Elevated plus maze test (EPM)

The EPM test was performed as previously described. The EPM consisted of a cross-shaped platform with two open arms and two closed arms (25 cm × 8 cm × 12 cm), elevated 50 cm above the ground. At the beginning of the experiment, the mice were placed in the central area facing the closed arm and allowed to explore freely for 5 min, and the activity was recorded using a camera located above the box. The time and number of entries in the open and closed arms were recorded.

#### 2.6.3 Fear extinction task (FET)

The FET test was performed as previously described (Gao et al., 2023). The fear extinction experiment was conducted using a combination of a neutral tone (CS) and a plantar shock (US), wherein the CS condition (1 kHz, 90 dB) remained constant, whereas the US condition (1 mA, 2 s) was administered via a metal mesh at the base of the fear chamber. The experiment comprised four stages. The initial stage involved an adaptation period on day one, during which the mice were placed in the apparatus for 5 min without any stimulation to eliminate the influence of unfamiliar environments. On day two, the mice were again placed in the fear chamber for 5 min with simultaneous application of both CS and US. US was administered three times, with intervals between each repetition lasting approximately 73–74 s. On day three, mice freely explored the fear chamber for 15 min under only CS conditions, and their freezing time was recorded at 3-min intervals. Finally, on day four, the freezing time of the mice in the fear chamber under CS conditions was recorded for 5 min to evaluate the regression of their fear memory.

$$\text{Extinction coefficient}=\left(1-\left(\text{Fear test after }24\;h\text{ freezing time}/\text{first }3\min\text{block freezing time}\right)\right)\times 100$$

### 2.7 Golgi staining and sholl analysis

Golgi staining was conducted utilizing the Golgi staining kit (Catalog No. G1152, Servicebio Technology, Wuhan, China). The mice were placed in a gas anesthesia chamber containing isoflurane and anesthetized until the absence of pain reflexes, followed by decapitation. The excised brain was rinsed with double-distilled water, subsequently immersed in a pre-prepared staining solution (comprising equal proportions of liquid A and liquid B), stored in the dark at room temperature for 14 days. Thereafter, it was transferred to solution C for 7 days before being sectioned into 100-micron coronal slices using an oscillating microslicer. The sections were mounted on gelatin slides and incubated in a mixture of D and E for 10 min. Following cleansing with double-distilled water, dehydration was performed using a series of ethanol concentrations, followed by three xylene washes prior to cover glass application. Subsequently, the Fiji software was used to trace and analyze the dendrites of pyramidal neurons in the CA1 region of the hippocampus. Sholl analysis in Fiji involved plotting concentric circles at equidistant intervals around the neuronal soma as a reference point to quantify the number of dendrites intersecting each circle. Add total dendrite length and branch count were also recorded.

### 2.8 Western blot

In the experimental protocols, after the mice were anesthetized, the hippocampal tissue was dissected under ice bath conditions, and then the tissue was typically subjected to lysis in RIPA buffer supplemented with protease and phosphatase inhibitors (manufactured by Shenzhen Solaibao Technology Co., Ltd.), and the protein concentration was determined using the BCA protein content determination kit (product number: ZJ102, produced by Nanjing Epizan Biotechnology Co., Ltd.). Subsequently, the proteins were separated via SDS-PAGE and transferred to a PVDF membrane. The membrane was then incubated in a 5% non-denatured milk powder solution at room temperature, followed by incubation with the primary antibody at 4°C overnight. The membrane was then washed four times with TBST for 5 min each. Thereafter, the membrane was incubated with the secondary antibody (Goat Anti-Rabbit/Mouse IgG, product number: ZB-2305, dilution ratio: 1:5,000, manufactured by Zhongshan Jinqiao Biotechnology Co., LTD.) at room temperature for 2 h and subjected to further washing with TBST buffer. Finally, protein visualization was conducted using the AI-800 imaging system (General Electric Company, United States), and the optical density values of each band were quantitatively analyzed using ImageJ software.

Primary antibody with the following proteins: anti-p-PKA (Ser133, 5661, 1:1,000, Cell Signaling Technology); anti-PKA (ab32514, 1:1,000, Abcam); anti-p-CREB (Ser133, ab32096, 1:1,000, Abcam); manti-CREB (ab32515, 1:1,000, Abcam); anti-BDNF (ab108319, 1:1,000, Abcam); anti-p-TrkB (ab229908, 1:1,000, Abcam); anti-TrkB (ab187041, 1:2,000, Abcam); anti-PSD95 (20665-1-AP, 1:1,000, Proteintech); anti-Synaptophysin (36406S, 1:1,000, Cell Signaling Technology); anti-NR2A (ab124913, 1:1,000, Abcam); anti-NR2B (ab65783, 1; 1,000, Abcam); anti-GluA1 (13185S, 1:1,000, Cell Signaling Technology); anti-GluA2 (13607S, 1:1,000, Cell Signaling Technology); anti-β-tubulin (AC021, 1:5,000, ABclonal); anti-GAPDH (60004-1-Ig, 1:5,000, Proteintech).

### 2.9 Statistical analysis

The experimental data were analyzed using GraphPad Prism 9.0 software, and statistical plots were expressed as mean ± SEM. For normally distributed data, independent sample t-test were used for two groups, and one-way or two-way ANOVA for four groups. Only significant comparisons are labeled in the figures. Statistical significance was set at P < 0.05.

## 3 Results

### 3.1 Activation of the cAMP-PKA ameliorated the deficits in fear extinction and anxiety-like behaviors in PTSD mice with alcohol exposure

**FIGURE 1:**
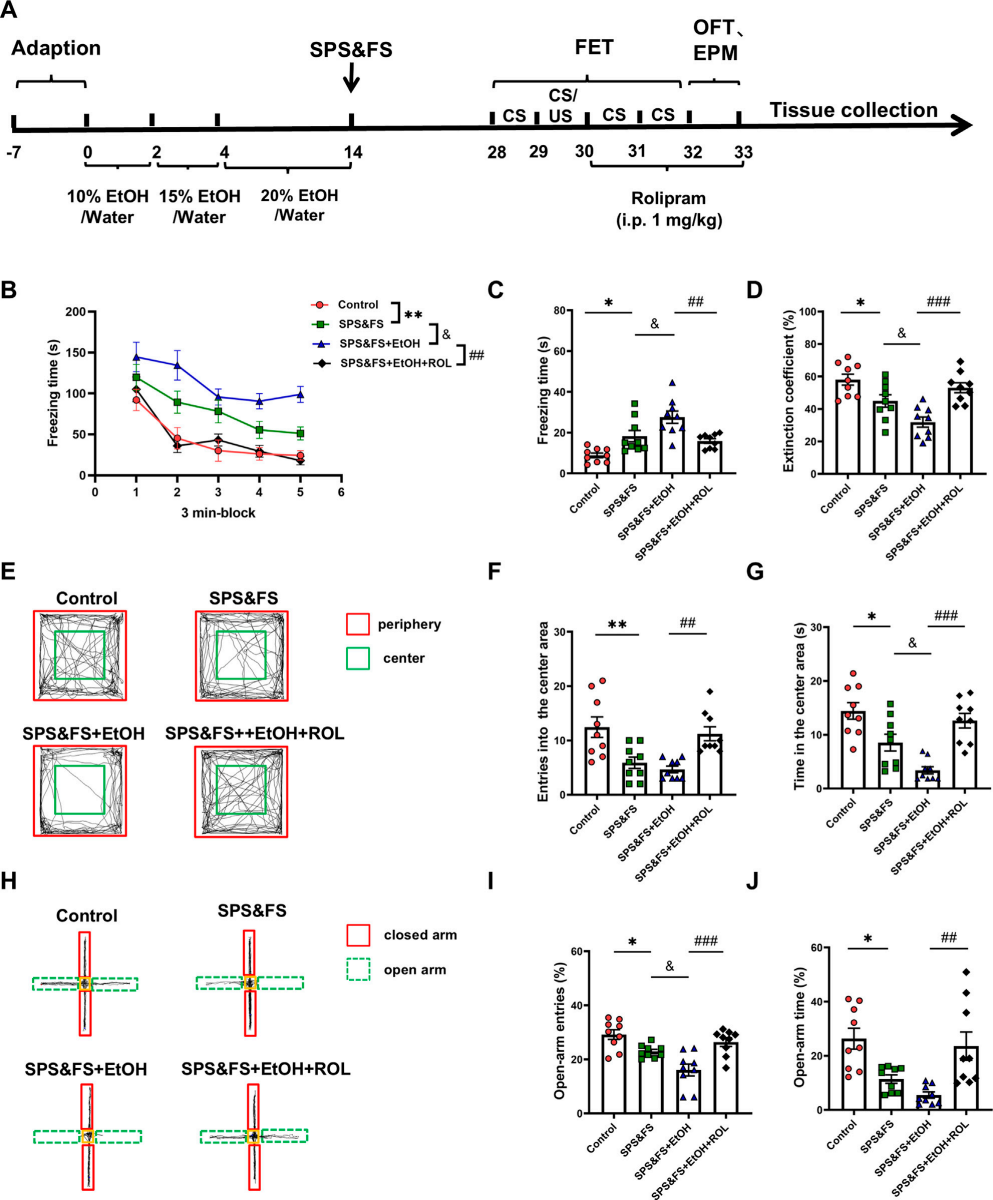
Activation of the cAMP-PKA ameliorated the deficits in fear extinction and anxiety-like behaviors in PTSD mice with alcohol exposure. **(A)** Flow chart of the experiment. **(B)** Freezing time during the re-exposure phase of FET. **(C)** Freezing time in the 24-h post-fear test. **(D)** The extinction coefficient measured in the FET test. **(E)** Representative tracking plot from the OFT. **(F, G)** Number of entries and time spent in the center area. **(H)** Representative track plot of the EPM test. **(I, J)** Number of entries and time spent in the open arms during the EPM test (n = 9 mice per group). All data are presented as mean ± SEM. Data were analyzed using contrast analyses following one-way or two-way ANOVA. *p < 0.05, **p < 0.01 vs. control; ^&^p < 0.05 vs. SPS&FS; ^##^p < 0.01, ^###^p < 0.001 vs. SPS&FS + EtOH.

To investigate the impact of early alcohol consumption on PTSD-like behavior and the effects of cAMP-PKA activation on fear extinction, we employed rolipram, a selective PDE4 inhibitor that upregulates cAMP-PKA pathway activity, developed the following experimental protocol (Figure 1A). The results showed that SPS&FS mice with prior alcohol exposure exhibited significantly impaired fear extinction, characterized by prolonged freezing time and reduced extinction coefficient [Figure 1B: F (3, 160) = 18.32, P < 0.0001; Figure 1C: F (3, 32) = 12.29, P < 0.0001; Figure 1D: F (3, 32) = 11.39, P < 0.0001]. Rolipram administration effectively enhanced fear memory extinction by reducing freezing time and increasing the extinction coefficient (Figures 1B–D). SPS&FS mice with prior alcohol exposure also exhibited more severe anxiety-like behavior, as evidenced by a significant decrease in entries [Figure 1F: F (3, 32) = 8.919, P = 0.0002; Figure 1G: F (3, 32) = 13.54, P < 0.0001] and time spent in the central area during the OFT and in the open arms during the EPM test [Figure 1I: F (3, 32) = 11.40, P < 0.0001; Figure 1J: F (3, 32) = 8.555, P = 0.0003]. Rolipram effectively alleviated anxiety-related phenotypes (Figures 1E–J). These findings highlight that alcohol exposure exacerbates fear extinction deficits and anxiety-like behavior in PTSD mice, with the significant ameliorating effects of rolipram on these behaviors.

### 3.2 Activation of cAMP-PKA ameliorates the impairment of hippocampal BDNF-TrkB signaling in PTSD mice with alcohol exposure

**FIGURE 2:**
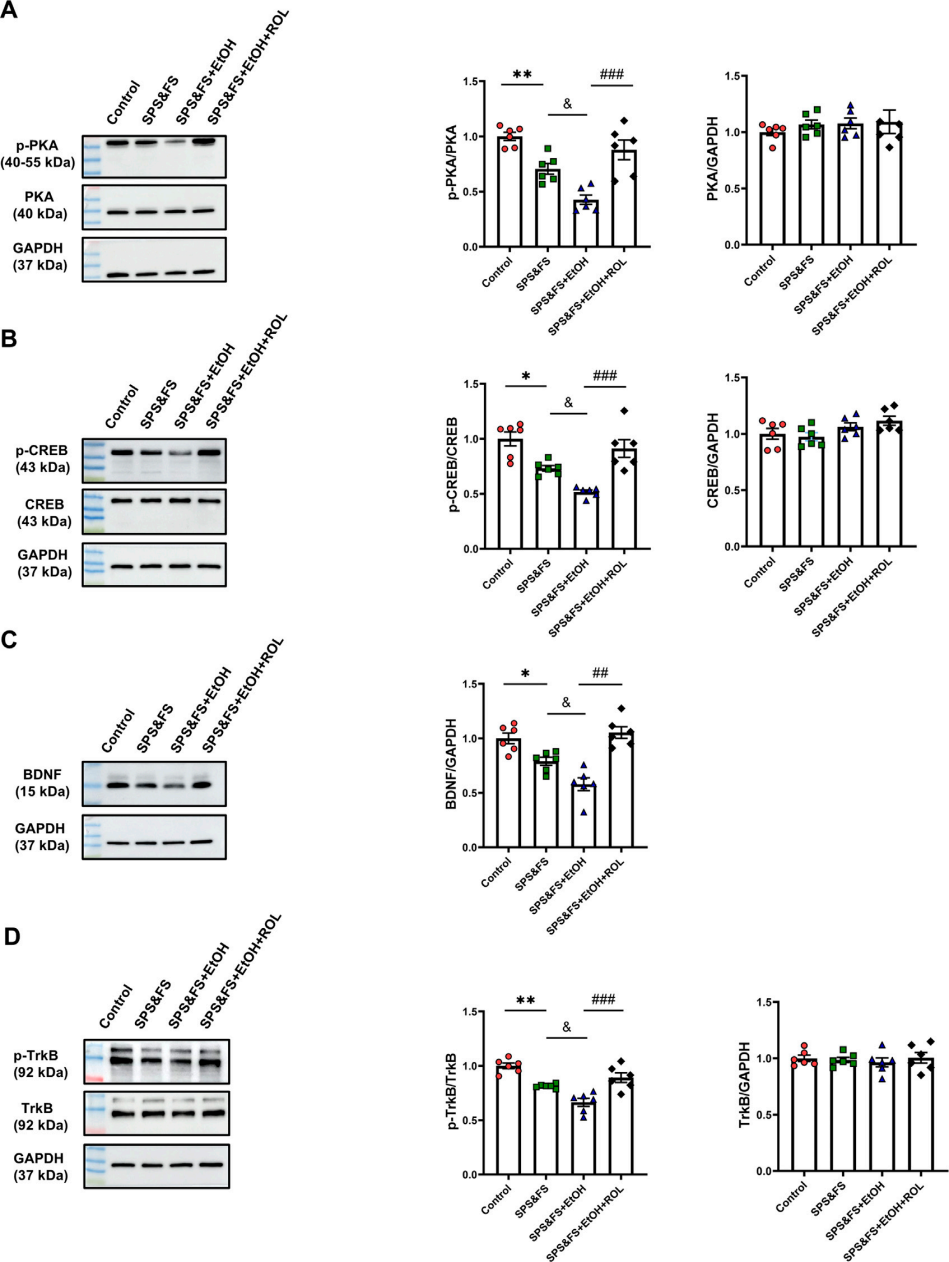
Activation of cAMP-PKA ameliorates the impairment of hippocampal BDNF-TrkB signaling in PTSD mice with alcohol exposure. **(A)** Protein expression of p-PKA, and PKA were detected by Western blot analysis. **(B)** Protein expression of p-CREB, and CREB was detected by Western blot analysis. **(C)** Protein expression of BDNF was detected by Western blot analysis. **(D)** Protein expression of p-TrkB, and PKA were detected by Western blot analysis (n = 6 mice per group). All data are presented as mean ± SEM. Data were analyzed using contrast analyses following one way ANOVA. *p < 0.05, **p < 0.01 vs. control; ^&^p < 0.05 vs. SPS&FS; ^##^p < 0.01, ^###^p < 0.001 vs. SPS&FS + EtOH.

In the central nervous system, PKA activation promotes CREB phosphorylation, influencing the transcription of target genes, including BDNF. This process contributes to the extinction of fear memory. To determine the role of the PKA signaling pathway in alcohol-exacerbated PTSD-like behavior, we detected changes in PKA, CREB, BDNF, and TrkB protein levels using Western blotting. The results showed that SPS&FS combined with alcohol consumption decreased the phosphorylation levels of PKA and CREB. Notably, rolipram treatment significantly increased the phosphorylation levels of PKA and CREB [Figure 2A: p-PKA, F (3, 20) = 18.34, P < 0.0001; PKA, F (3, 20) = 0.4219, P = 0.7393; Figure 2B: p-CREB, F (3, 20) = 16.05, P < 0.0001; CREB, F (3, 20) = 2.593, P = 0.0811]. Additionally, alcohol exposure in SPS&FS mice led to a significant decrease in BDNF and TrkB phosphorylation levels. Administration of rolipram effectively restored these molecular deficits [Figure 2C: BDNF, F (3, 20) = 14.45, P < 0.0001; Figure 2D: p-TrkB, F (3, 20) = 19.57, P < 0.0001; TrkB, F (3, 20) = 0.2584, P = 0.8545]. These findings suggest that rolipram administration facilitates fear extinction and attenuates anxiety-like behavior by potentiating the PKA/CREB/BDNF/TrkB signaling pathway.

### 3.3 Activation of the cAMP-PKA mitigated the reduction in synaptic plasticity proteins in the hippocampus of PTSD mice with alcohol exposure

**FIGURE 3:**
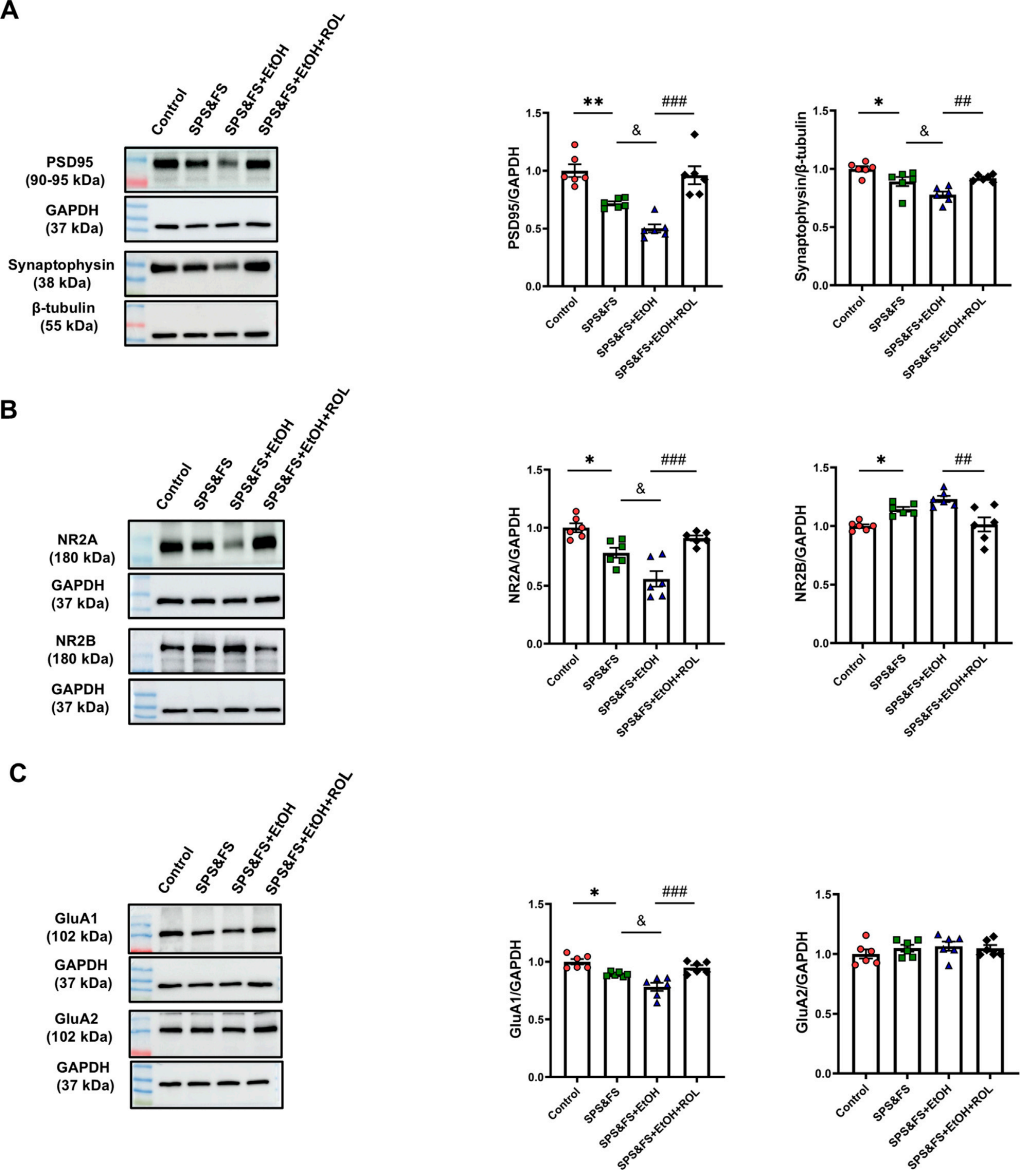
Activation of the cAMP-PKA mitigated the reduction in synaptic plasticity proteins in the hippocampus of PTSD mice with alcohol exposure. **(A)** Protein expression of PSD95, and Synaptophysin was detected by Western blot analysis. **(B)** Protein expression of NR2A, and NR2B were detected by Western blot analysis. **(C)** Protein expression of GluA1, and GluA2 was detected by Western blot analysis (n = 6 mice per group). All data are presented as mean ± SEM. Data were analyzed using contrast analyses following one-way or two-way ANOVA. *p < 0.05, **p < 0.01 vs. control; ^&^p < 0.05 vs. SPS&FS; ^##^p < 0.01, ^###^p < 0.001 vs. SPS&FS + EtOH.

Considering the key regulatory role of BDNF in synaptic plasticity, we further investigated the expression of other key synaptic structure-related proteins to provide a more comprehensive elucidation of the underlying molecular mechanisms involved. The results showed that the expression of PSD95 and synaptophysin was significantly decreased in SPS&FS mice after exposure to alcohol. Rolipram treatment significantly attenuated these impairments [Figure 3A: PSD95, F (3, 20) = 17.19, P < 0.0001; synaptophysin, F (3, 20) = 11.49, P = 0.0001]. In addition, we studied the expression of AMPA and NMDA receptor subunits. The data showed that NR2A expression was downregulated, whereas NR2B expression was upregulated in SPS&FS mice with early alcohol consumption. These phenotypic changes were reversed by the administration of rolipram [Figure 3B: NR2A, F (3, 20) = 17.50, P < 0.0001; NR2B, F (3, 20) = 9.738, P = 0.0004]. GluA1 and GluA2 are the two main subunits of the AMPA receptor. In alcohol-exposed SPS&FS mice, the protein levels of GluA1 were significantly reduced, whereas those of GluA2 remained unchanged. Rolipram treatment effectively restored GluA1 expression to normal levels [Figure 3C: GluA1, F (3, 20) = 15.28, P < 0.0001; GluA2, F (3, 20) = 0.7103, P = 0.5572]. These findings provide further evidence that modulating synaptic plasticity via PKA contributes to the improvement of PTSD-like behaviors.

### 3.4 Activation of the cAMP-PKA ameliorated the impairment of dendritic structure in hippocampal CA1 pyramidal cells in PTSD mice with alcohol exposure

**FIGURE 4:**
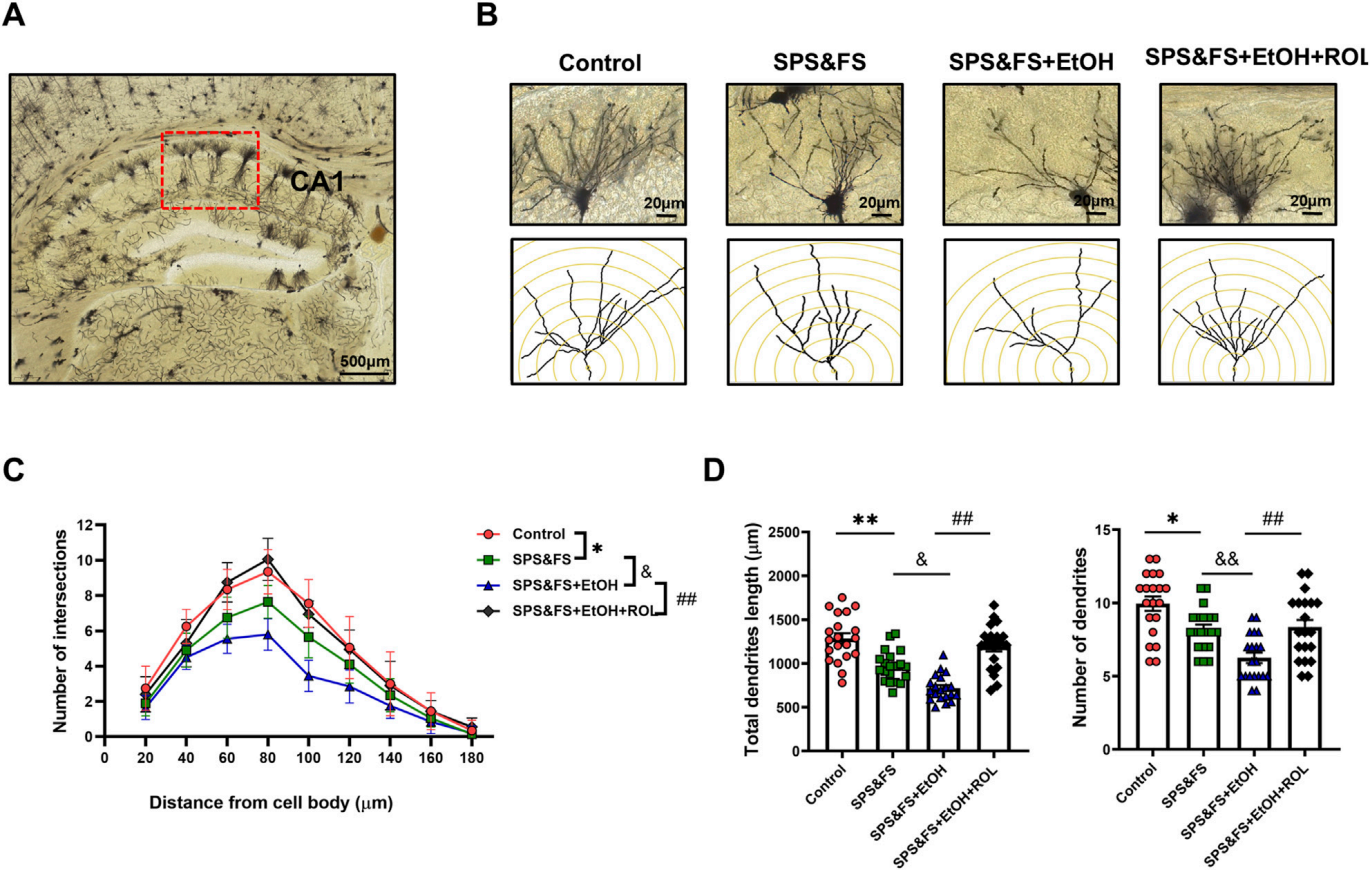
Activation of the cAMP-PKA ameliorated the impairment of dendritic structure in hippocampal CA1 pyramidal cells in PTSD mice with alcohol re-exposure. **(A)** Representative images of the hippocampal CA1 region labeled using Golgi staining. Scale bar = 500 μm. **(B)** Representative images of neuronal dendrites. Scale bar = 20 μm. **(C)** Sholl analysis of the hippocampal CA1 neurons revealed the alterations of basal dendritic intersections at distinct distances from soma (n = 20 cells/five mice per group). **(D)** Total dendritic length and dendritic numbers of neurons (n = 9 mice per group). All data are presented as mean ± SEM. Data were analyzed using contrast analyses following one-way ANOVA. *p < 0.05, **p < 0.01 vs. control; ^&^p < 0.05, ^&&^p < 0.01 vs. SPS&FS; ^##^p < 0.01, vs. SPS&FS + EtOH.

We detected changes in synaptic proteins at the molecular level and subsequently examined the morphological structure of the synapse. The CA1 subregion of the hippocampus, which is critical for long-term potentiation and structural plasticity, was stained using the Golgi technique to visualize the complexity of dendrites. Representative images and reconstructed dendritic structures are shown in Figures 4A, B. Concentric circles spaced at 20 μm intervals were drawn, and the number of intersections between the concentric circles and dendrites was calculated as a parameter to evaluate the complexity of the dendritic tree. Sholl analysis revealed that the number of intersections between the dendrites and concentric circles varied at different radial distances from the cell body [Figure 4C: F (3, 684) = 16.35, P < 0.0001]. Notably, the number of dendritic intersections was significantly reduced in alcohol-exposed SPS&FS mice. This reduction was markedly attenuated by rolipram treatment, leading to the restoration of levels comparable to those in the control group. Rolipram also effectively prevented the alcohol-induced reduction in total dendrite length and overall dendritic numbers [Figure 4D; total dendrite length, F (3, 76) = 9.149, P < 0.0001; dendritic numbers, F (3, 76) = 12.81, P < 0.0001]. These results suggest that rolipram treatment ameliorated the impairment of dendritic structures in the hippocampal CA1 region of PTSD mice with alcohol exposure.

### 3.5 Activation of the cAMP-PKA attenuated alcohol consumption and preference in PTSD mice with alcohol exposure

**FIGURE 5:**
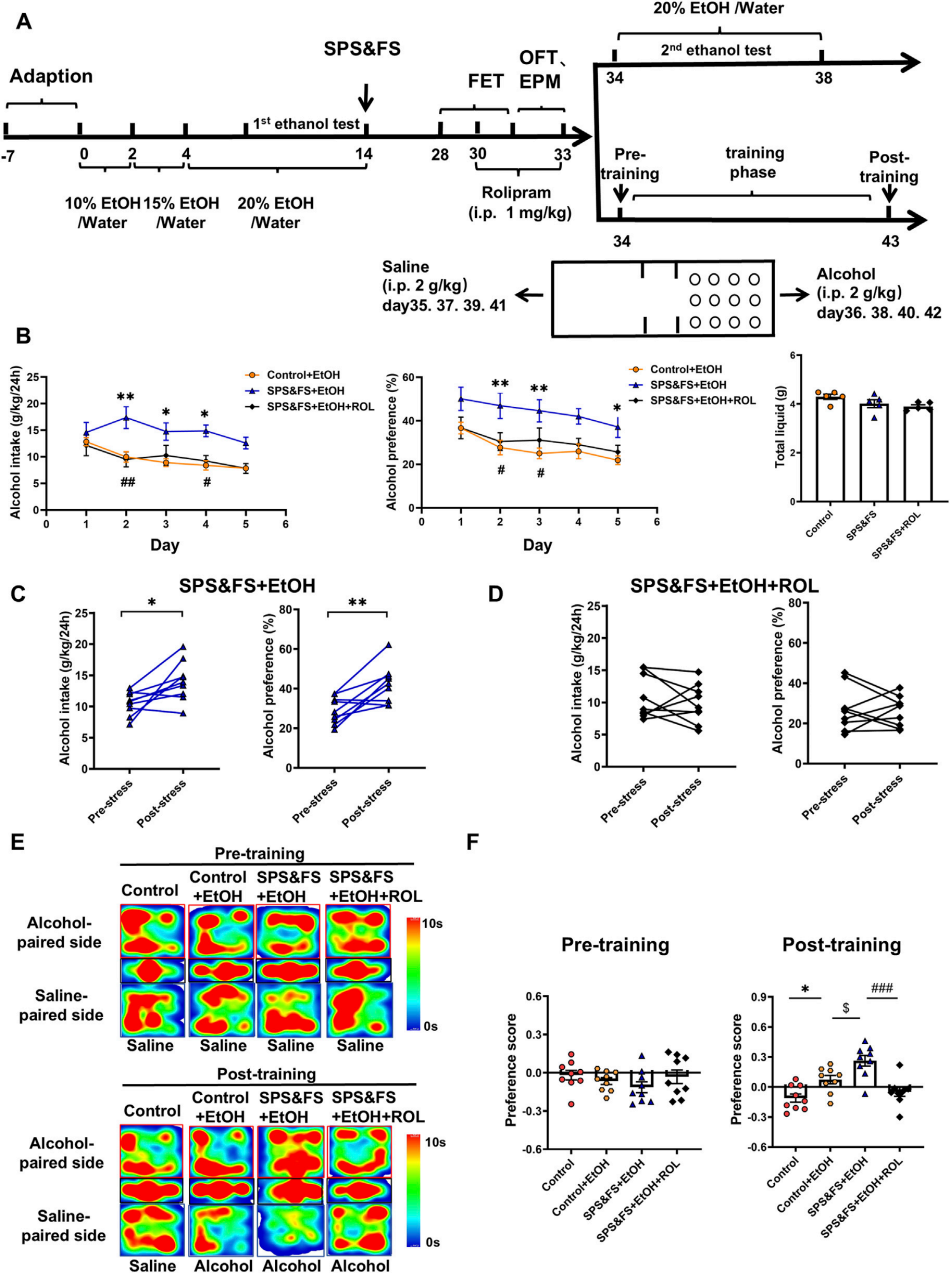
Activation of the cAMP-PKA attenuated alcohol consumption and preference in PTSD mice with alcohol exposure. **(A)** Flow chart of the experiment. **(B)** Alcohol intake, alcohol preference and total liquid were measured during rolipram treatment. **(C)** Paired t-tests were used to detect the changes in alcohol intake and preference in the same mice before and after exposure to stress. **(D)** Paired t-tests were conducted to compare alcohol intake and preference in the same mice before and after exposure to stress, following rolipram treatment. **(E)** Heatmap representation of the mouse movement trajectory during the CPP test. **(F)** Preference scores in CPP test were detected. All data are presented as mean ± SEM. Data were analyzed using contrast analyses following one-way or two-way ANOVA. *p < 0.05, **p < 0.01 vs. control; ^$^p < 0.05 vs. control + EtOH; ^#^p < 0.05, ^##^p < 0.01, ^###^p < 0.001 vs. SPS&FS + EtOH.

To evaluate the effects of SPS&FS traumatic stress on AUD-related behaviors and the regulatory role of the PKA signaling pathway. We conducted 2-BC and CPP experiments at later stages (Figure 5A). Prior to the application of SPS&FS stimulation, all mice exhibited comparable levels of alcohol consumption and preference, with no significant distinctions observed between the experimental groups [Supplementary Figure S1A: alcohol intake, F (2, 120) = 1.086, P = 0.3409; alcohol preference, F (2, 120) = 2.321, P = 0.1025; total liquid, F (2, 12) = 0.9157, P = 0.4265]. The comparison between SPS&FS mice and non-stressed mice showed that the comorbidities exacerbated excessive drinking behavior, characterized by increased alcohol intake and preference, while total fluid consumption remained unaffected [Figure 5B: F (2, 12) = 2.874, P = 0.0955]. However, treatment with rolipram significantly attenuated this stress-induced escalation in alcohol consumption [Figure 5B: alcohol intake, F (2, 130) = 22.41, P < 0.0001; alcohol preference, F (2, 120) = 23.10, P < 0.0001]. To facilitate a more precise observation of alcohol consumption, we used a paired t-test to compare alcohol intake and preference in the same group of mice before and after exposure to SPS&FS stress, as well as before and after rolipram administration. The analysis revealed a substantial and statistically significant increase in alcohol consumption in mice following exposure to SPS&FS stress [Figure 5C: Alcohol intake, t (8) = 2.851, P = 0.0214; Alcohol preference, t (8) = 3.794, P = 0.0053]. Notably, the administration of rolipram effectively prevented any further escalation in alcohol intake or preference in stress-exposed mice [Figure 5D: alcohol intake, t (8) = 0.8249, P = 0.4333; alcohol preference, t (8) = 0.2298, P = 0.8240].

The alcohol-induced CPP assay was used to assess alcohol preference in SPS&FS mice both before and after rolipram administration. After training, environmental cues elicited alcohol craving and seeking. During the pre-training process, the mice in each group did not show a clear preference for either side of the box. After training, stressed mice showed a marked preference for the alcohol-paired side, spending significantly more time there and achieving higher preference scores. After PKA activation by rolipram, the stressed mice showed a significant reduction in preference and time spent on the alcohol-paired side, indicating a decrease in alcohol preference after PTSD stress [Figures 5E, F: Pre-training, F (3, 32) = 1.065, P = 0.3778; Post-training, F (3, 32) = 13.14, P < 0.0001].

## 4 Discussion

**FIGURE 6:**
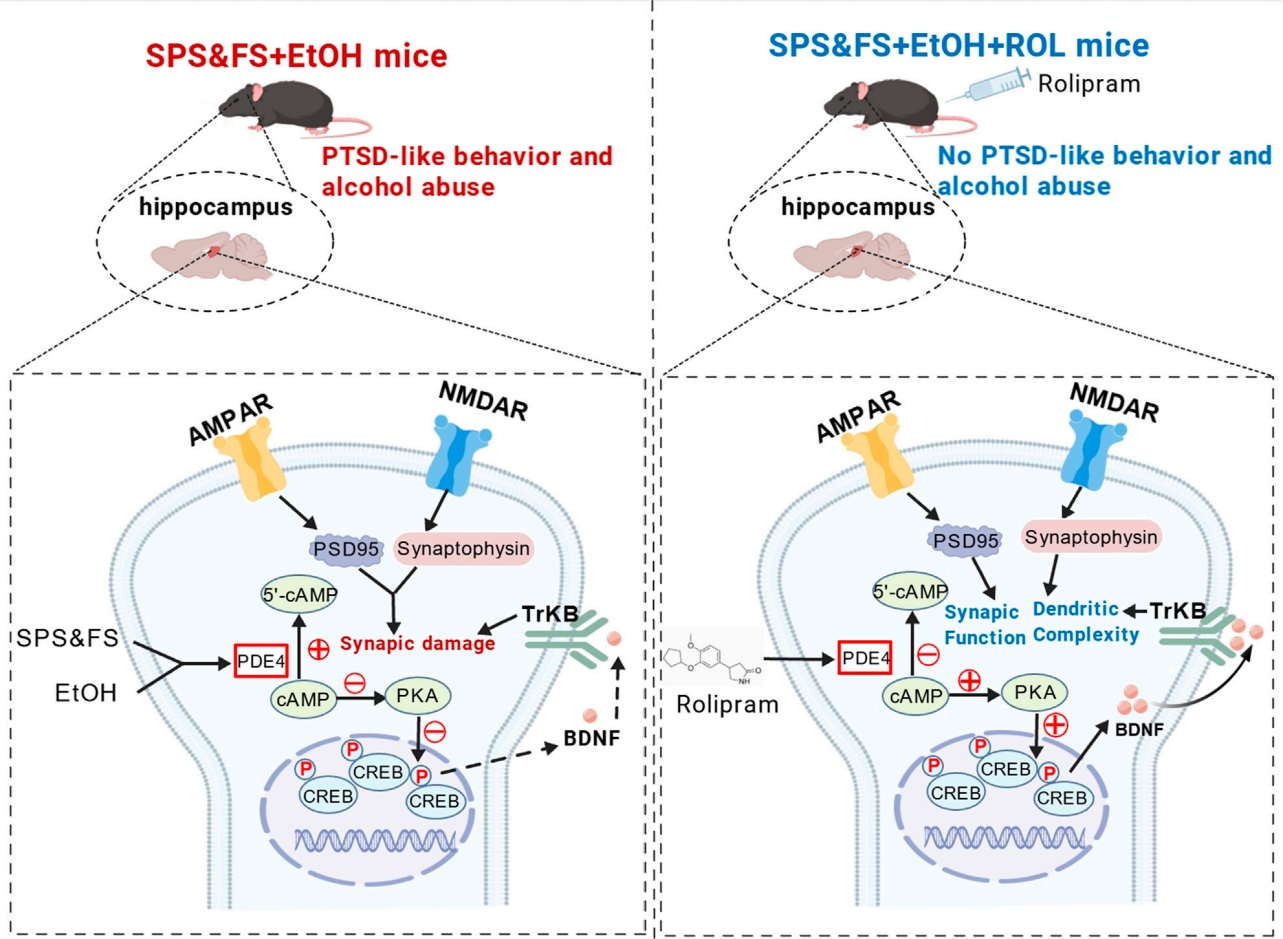
Mechanistic illustration of rolipram-induced cAMP-PKA activation in the therapeutic of PTSD-like behaviors exacerbated by alcohol exposure. Alcohol exposure together with later SPS&FS trauma stimulation promoted cAMP hydrolysis, downregulated the activity of PKA-CREB-BDNF-TrkB signaling, resulting in aberrant expression of the key synaptic protein AMPA with NMDA receptor subunits, PSD95 and Synaptophysin, and this synaptic impairment exacerbated fear and anxiety-like behavior in PTSD mice to induce AUD-related behaviors. Following Rolipram administration, the PKA-CREB-BDNF-TrkB signaling pathway was activated, leading to the upregulation and downregulation of synaptic-associated proteins. This activation enhanced synaptic structure and function, as well as dendritic spine complexity, thereby ameliorating fear extinction deficits and anxiety-like behaviors in PTSD mice with alcohol exposure. Additionally, it reduced ethanol intake and alcohol preference in mice during subsequent alcohol re-.exposure.

The interaction between alcohol and stress is complex, with stress serving as the primary driver of relapse following abstinence (Breese et al., 2011; Fein, 2013). Alcohol significantly affects various learning and memory processes, including fear conditioning (Alijan-Pour et al., 2012). Furthermore, chronic substance use exacerbates arousal levels, anxiety, and sensitivity of neurobiological stress systems, thereby elevating the risk of PTSD development (Sinha, 2024). Consequently, alcohol use may substantially increase individual susceptibility to PTSD. In this study, we found that individuals with early alcohol exposure showed more pronounced fear extinction deficits and higher anxiety levels, and that the administration of rolipram to activate the cAMP-PKA signaling pathway effectively ameliorated these impairments. Our study confirmed that activating the PKA pathway to regulate synaptic plasticity can alleviate fear extinction deficits and reduce alcohol preference following traumatic stress. Rolipram treatment reversed the downregulation of BDNF and TrkB, increased the expression of PSD95 and synaptophysin, improved the abnormal expression of AMPA and NMDA receptor subtypes, and rescued damage to synaptic structures (Figure 6). This study provides crucial insights into the modulation of synaptic plasticity in the hippocampus through cAMP-PKA pathway activation, providing potential therapeutic strategies for PTSD/AUD treatment.

Extensive clinical research has consistently identified alcohol consumption as a significant barrier to recovery in patients with various psychiatric disorders. Early exposure to alcohol not only increases susceptibility to PTSD, but also exacerbates its severity (Possemato et al., 2022; McFarlane et al., 2009). While existing studies have predominantly focused on the influence of stress on subsequent alcohol consumption patterns, emerging evidence suggests that early alcohol exposure exacerbates the traumatic phenotype in mouse models of PTSD (Anderson et al., 2016; Gonzalez et al., 2021; Barkell et al., 2022). However, the precise neural mechanisms underlying this phenomenon remain poorly understood. To address this critical gap, we established a novel model by sequentially exposing mice to ethanol followed by traumatic stimulation. Comprehensive behavioral analyses revealed that PTSD mice with prior alcohol exposure exhibited significant deficits in fear memory extinction and pronounced anxiety-like behaviors, thereby validating the detrimental effects of ethanol exposure on stress response.

Traumatic memory represents a central component in the pathophysiology of PTSD, with extinction serving as a fundamental mechanism for attenuating trauma-associated memories (Kida, 2019). Emerging evidence suggests that enhancing the fear extinction process through pharmacological assistance can significantly improve the effect of PE therapy and effectively shorten the duration of treatment (Maren and Holmes, 2016). This underscores the need for further investigation into the regulatory mechanisms underlying fear resolution and the identification of more potent therapeutic targets. Recent experimental studies emphasize the importance of enhanced hippocampal neurogenesis in facilitating the extinction of fear memories. Building on this finding, we investigated the potential implications of hippocampal synaptic plasticity in the context of alcohol exposure, which exacerbates PTSD-like behaviors.

The cAMP-PKA pathway plays a crucial role in fear extinction, mainly by modulating hippocampal synaptic plasticity via BDNF/TrkB signaling (Gao et al., 2023; Ji et al., 2023; Chen et al., 2024). TrkB, the specific receptor for BDNF, is widely distributed in the hippocampal dendritic spines and pyramidal neurons. The binding of BDNF to TrkB initiates persistent signaling in dendrites and axons, thereby regulating dendritic development (Qiu et al., 2020). Disruption of the BDNF/TrkB signaling pathway leads to a substantial decrease in both early and late LTP at CA1 hippocampal synapses, ultimately impairing the memory function associated with synaptic activity (Moya-Alvarado et al., 2023). In our study, we observed a significant downregulation of key proteins in the PKA/BDNF/TrkB pathway. Golgi staining revealed reduced dendritic complexity and length in hippocampal CA1, underscoring the critical role of this pathway in the pathophysiology of alcohol exposure-induced exacerbation of PTSD-like behaviors, as well as its contribution to fear extinction deficits and anxiety-like behaviors.

PSD95, a critical scaffold protein essential for the organization and function of the postsynaptic density complex, plays a pivotal role in synaptic plasticity (Zeng et al., 2016). Synaptophysin, a synaptic vesicle membrane protein, is another key regulator of synaptic activity (Marco-Manclus et al., 2022). Together with key glutamate-gated ion channels, such as NMDAR and AMPAR, they regulate synaptic plasticity and mediate neurodegenerative and neuroprotective processes in the brain. NR2A, a predominant subunit of NMDARs, is predominantly localized at synaptic sites, where it facilitates CREB activation and subsequent expression of plasticity-related genes (Scherzer et al., 1998; Law et al., 2003; Ji et al., 2017). In contrast, NR2B subunits are primarily expressed in extra synaptic regions, where their activation leads to sustained CREB dephosphorylation and transcriptional inactivation (Gong et al., 2020). Our analysis revealed significant downregulation of PSD95, synaptophysin, and NR2A and upregulation of NR2B expression in SPS&FS mice exposed to alcohol. Rolipram treatment effectively attenuated these reductions and restored the protein levels. Regarding AMPA receptor subunits, we focused on GluA1 and GluA2, which are the two principal components of this receptor complex (Stockwell et al., 2024). Alcohol-exposed SPS&FS mice exhibited significant reductions in GluA1 protein levels, whereas GluA2 expression remained unchanged. Rolipram treatment effectively restored GluA1 expression to normal levels.

Stress-induced ethanol consumption in mice is mediated by PTSD-related anxiety and alcohol exposure (Pucci et al., 2019; Khantzian, 1997). Our study revealed that mice with PTSD and early alcohol consumption displayed elevated anxiety-like behavior. Sequential alcohol exposure, trauma induction, and subsequent alcohol reintroduction significantly increased ethanol consumption and dependence, as evidenced by the CPP test. Rolipram-induced activation of the PKA pathway effectively mitigated these behavioral and physiological effects, highlighting the role of alcohol in stress-related consumption.

In conclusion, this study demonstrates that activation of the PKA signaling pathway regulates the expression of AMPA and NMDA receptor subunits, enhances synaptic structure and function in the hippocampus, and improves the fear response and drinking behavior in exacerbated PTSD-like behaviors due to alcohol exposure. These findings suggest that synaptic plasticity may be the underlying neural mechanism for the comorbidity of alcohol exposure and exacerbated PTSD-like behaviors. This could explain the intensification of PTSD-like behavior after early alcohol consumption. Therefore, targeting synaptic plasticity via the PKA pathway may represent a promising therapeutic strategy for treating comorbid PTSD and AUD.

## 5 Conclusion

Prior alcohol exposure exacerbates fear extinction deficits and intensifies anxiety-like behaviors in murine models of PTSD disorder. These detrimental effects can be effectively mitigated through the activation of the PKA signaling pathway, which enhances hippocampal synaptic function and reduces alcohol consumption and preference following a traumatic stress. Consequently, therapeutic modulation of synaptic plasticity through the PKA pathway may represent a promising novel intervention strategy for treating comorbid PTSD and AUD in the future.

## 6 Limitations

The relationship between early alcohol exposure and PTSD in female mice was not explored. 2) Our study focused only on the hippocampus and did not consider the role played by other brain regions. In addition to the hippocampus, the prefrontal cortex is also involved in the control of fear memory, and the cerebral cortex and hippocampus may cooperate to control fear. 3) Rolipram’s modulation of synaptic plasticity may involve multiple pathways, future studies could benefit from targeted genetic or optogenetic manipulations to confirm cAMP-PAK signaling pathway specificity.

## Data Availability

The raw data supporting the conclusion of this article will be made available by the authors, without undue reservation.
