# Supplementary material for: The regulation and mechanism of the cAMP-PKA pathway on PTSD-like behaviors exacerbated by alcohol exposure
Source: Front Pharmacol. 2025 May 16;16:1592187. doi: 10.3389/fphar.2025.1592187 (PMC12122473; doi:10.3389/fphar.2025.1592187)
Supplement: Supplementary file 1 [file Image1.pdf]

Supplementary Material

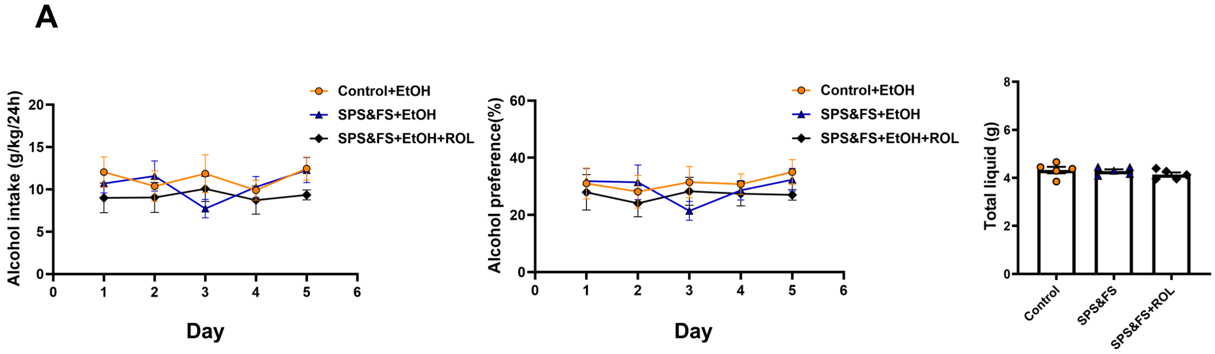

Supplementary Figure 1 There were no differences in alcohol consumption among the groups prior to SPS&FS. (A) Alcohol intake, alcohol preference and total liquid was detected before SPS&FS stress. All data are presented as mean ± SEM. Data were analyzed using contrast analyses following or one-way or two-way ANOVA. n = 9 mice per group.
